# Complete Nucleotide Sequence of Canine Distemper Virus HLJ1-06, Isolated from Foxes in China

**DOI:** 10.1128/genomeA.00065-12

**Published:** 2013-01-31

**Authors:** Qian Jiang, Xiaoliang Hu, Yanhua Ge, Huan Lin, Yong Jiang, Jiasen Liu, Dongchun Guo, Changde Si, Liandong Qu

**Affiliations:** Division of Laboratory Animals, National Key Laboratory of Veterinary Biotechnology, Harbin Veterinary Research Institute, Chinese Academy of Agricultural Sciences, Harbin, People’s Republic of China

## Abstract

A new strain of canine distemper virus, HLJ1-06, has been isolated from foxes in China, and its complete genome has been sequenced and analyzed. The phylogenetic analysis suggests that HLJ1-06 belongs to the Asia-1 cluster and has low identity to the vaccine strain.

## GENOME ANNOUNCEMENT

Canine distemper virus (CDV), which belongs to the genus *Morbillivirus* in the *Paramyxoviridae* family, possesses a single-stranded negative RNA encoding six nonoverlapping transcriptional units which produce eight proteins (1). Long known to cause potentially lethal disease among members of the *Canidae*, *Mustelidae*, and *Procyonidae*, CDV has recently been detected as a cause of morbidity and mortality in large felids (2), freshwater seals (*Phoca sibirica*) (3), and various other animals. There is one serotype, and seven different genotypes have been described (Asia-1, Asia-2, Europe, European wildlife, Arctic-like, America-1, and America-2). A vaccine strain of CDV, Onderstepoort, which is widely used, belongs to America-1. One case of suspicion of CDV infection was identified initially by the animal owner on the basis of the clinical signs (diarrhea, cough, fever, nasal discharge, myoclonia, and nasal and foot-pad hyperkeratosis). Tissue samples were analyzed with a commercial CDV kit (Bioindist, South Korea) and reverse transcription (RT)-PCR by using a primer pair (forward, ATCATAGACGACCCTGATG; reverse, GACCCTTCGTCTACAATTTTG) that amplified a 144-bp-long fragment of the N gene, which indicated that the infecting agent was CDV. The viral RNA was extracted from tissue samples using Trizol reagent (Invitrogen) according to the manufacturer’s instructions. The first-strand cDNA was synthesized from viral RNA using a random primer (Invitrogen). Thirteen pairs of primers were designed from the published sequences of CDV strains A75/17 (GenBank accession no. AF164967), 5804 (GenBank accession no. AY386315), and Onderstepoort (GenBank accession no. AF305419) to amplify most of the HLJ1-06 strain genome except the 3′ and 5′ termini. The sequences of genome termini were determined by rapid amplification of cDNA ends (RACE) by using a 3′-full RACE kit and 5′-full RACE kit (TaKaRa). The PCR products were cloned into the pMD18-T vector (TaKaRa) and sequenced using an ABI 3730XL Sanger-based genetic analyzer. Sequences were compiled using the SEQMAN program in LASERGENE. The genome of the HLJ1-06 strain is 15,690 nucleotides (nt) and consists of six genes in the order of 3′ N-P-M-F-H-L 5′. The levels of identity between the complete genome sequence of the HLJ1-06 strain and those of 25 other CDVs ranged from 92.7% (for strain Onderstepoort) to 99% (for strain PS; GenBank accession no. JN896331.1). The levels of identity for the F gene ranged from 90.9% (for strain Onderstepoort) to 99.3% [for strain HeB(07)1; GenBank accession no. EU327874], and those for the H gene ranged from 91.2% (for strain Onderstepoort) to 99.4% [for strain SD(09)3; GenBank accession no. HM448834.1]. The phylogenetic analysis and multiple sequence alignment according to the nucleotide sequence of the H gene revealed that HLJ1-06 belongs to the Asia-1 cluster, which includes most Chinese strains. Nine potential N-linked glycosylation sites were found (amino acids [aa] 19 to 21, 149 to 151, 309 to 311, 391 to 393, 422 to 424, 456 to 458, 584 to 586, 587 to 589, and 603 to 605), four of which were identical to those in the Onderstepoort strain.

The complete sequence of the HLJ1-06 strain permitted a detailed analysis of the genetic variation of CDVs in China that is likely to be helpful to guide efficient diagnostic, preventive, and control strategies for CD in China. In addition, the data can be used to guide the selection of vaccine strains according to the variation of the H protein.

### Nucleotide sequence accession number.

The genome sequence of the HLJ1-06 strain has been submitted to GenBank (accession number JX681125).
